# Supplementary material for: Ursodeoxycholic acid and severe COVID-19 outcomes in a cohort study using the OpenSAFELY platform
Source: Commun Med (Lond). 2024 Nov 19;4:238. doi: 10.1038/s43856-024-00664-y (PMC11576861; doi:10.1038/s43856-024-00664-y)
Supplement: Supplementary file 2 — Supplementary materials [file 43856_2024_664_MOESM2_ESM.pdf]

Supplementary materials

Table of contents:

Supplementary Note 1: Information governance details 2

Supplementary Figure 1: Study population flowchart 3

Supplementary Table 1: Characteristics of those with PBC by ursodeoxycholic acid (UDCA) exposure status at index date 4

Supplementary Table 2: Characteristics of those with PSC by ursodeoxycholic acid (UDCA) exposure status at index date 5

Schoenfeld residual plots 6

- Supplementary Figure 2: Primary outcome: COVID-19 related hospitalisation or death 6
- Supplementary Figure 3: Secondary outcome: COVID-19 related hospitalisation 7
- Supplementary Figure 4: Secondary outcome: COVID-19 related death 8

Sensitivity analysis results 9

- Supplementary Table 3: 90 day exposure 9
- Supplementary Table 4: 120 day exposure with overlapping days added to next available unexposed period 10
- Supplementary Table 5: PBC population only 11
- Supplementary Table 6: PSC population only 12
- Supplementary Table 7: Exclusion of people prescribed obeticholic acid at baseline 13
- Supplementary Table 8: Exclusion of people with missing information for smoking status, BMI and ethnicity. 14
- Supplementary Table 9: Secondary analysis: Vaccinated cohort with index date 1st March 2021 15
- Supplementary Table 10: OpenSAFELY Collaborative 16
- Supplementary Table 11: The LH&W NCS Collaborative 17

Supplementary Note 1: Information governance details

Patient data has been pseudonymised for analysis and linkage using industry standard cryptographic hashing techniques; all pseudonymised datasets transmitted for linkage onto OpenSAFELY are encrypted; access to the NHS England OpenSAFELY COVID-19 service is via a virtual private network (VPN) connection; the researchers hold contracts with NHS England and only access the platform to initiate database queries and statistical models; all database activity is logged; only aggregate statistical outputs leave the platform environment following best practice for anonymisation of results such as statistical disclosure control for low cell counts (1).

The service adheres to the obligations of the UK General Data Protection Regulation (UK GDPR) and the Data Protection Act 2018. The service previously operated under notices initially issued in February 2020 by the the Secretary of State under Regulation 3(4) of the Health Service (Control of Patient Information) Regulations 2002 (COPI Regulations), which required organisations to process confidential patient information for COVID-19 purposes; this set aside the requirement for patient consent (2). As of 1 July 2023, the Secretary of State has requested that NHS England continue to operate the Service under the COVID-19 Directions 2020 (3). In some cases of data sharing, the common law duty of confidence is met using, for example, patient consent or support from the Health Research Authority Confidentiality Advisory Group (4).

Taken together, these provide the legal bases to link patient datasets using the service. GP practices, which provide access to the primary care data, are required to share relevant health information to support the public health response to the pandemic, and have been informed of how the service operates.

1. NHS Digital [Internet]. [cited 2023 Sep 20]. ISB1523: Anonymisation Standard for Publishing Health and Social Care Data. Available from: https://digital.nhs.uk/data-and-information/information-standards/information-standards-and-data-collections-including-extractions/publications-and-notifications/standards-and-collections/isb1523-anonymisation-standard-for-publishing-health-and-social-care-data
2. GOV.UK [Internet]. 2022 [cited 2023 Sep 20]. [Withdrawn] [withdrawn] Coronavirus (COVID-19): notice under regulation 3(4) of the Health Service (Control of Patient Information) Regulations 2002 – general. Available from: https://www.gov.uk/government/publications/coronavirus-covid-19-notification-of-data-controllers-to-share-information/coronavirus-covid-19-notice-under-regulation-34-of-the-health-service-control-of-patient-information-regulations-2002-general--2
3. NHS Digital [Internet]. [cited 2023 Sep 20]. COVID-19 Public Health Directions 2020. Available from: https://digital.nhs.uk/about-nhs-digital/corporate-information-and-documents/directions-and-data-provision-notices/secretary-of-state-directions/covid-19-public-health-directions-2020
4. Health Research Authority [Internet]. [cited 2023 Sep 20]. Confidentiality Advisory Group. Available from: https://www.hra.nhs.uk/about-us/committees-and-services/confidentiality-advisory-group/

Supplementary Figure 1: Study population flowchart

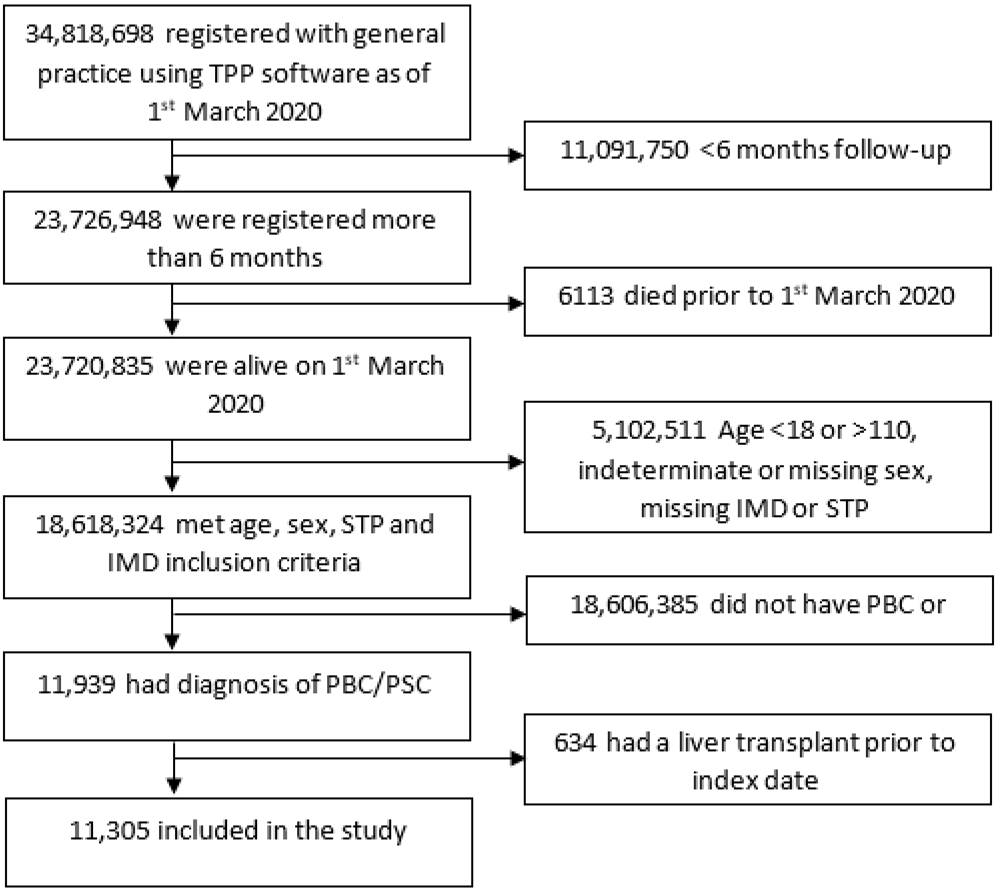

Supplementary Table 1: Characteristics of those with PBC by ursodeoxycholic acid (UDCA) exposure status at index date

Characteristic		Overall N=8805	No UDCA at baseline N=2780	UDCA at baseline N=6020
Age category	18 - 40 years	215 (2.4)	100 (3.6)	115 (1.9)
	41 - 60 years	2275 (25.8)	750 (26.9)	1525 (25.3)
	61 - 80 years	4900 (55.7)	1400 (50.4)	3500 (58.1)
	>80 years	1415 (16.1)	530 (19.1)	880 (14.7)
Sex	Female	7775 (88.3)	2475 (89)	5300 (88)
	Male	1025 (11.7)	305 (11)	720 (12)
Index of multiple deprivation	1 (Most deprived)	1705 (19.4)	595 (21.3)	1115 (18.5)
	2	1625 (18.5)	500 (18)	1125 (18.7)
	3	1945 (22.1)	605 (21.7)	1340 (22.3)
	4	1805 (20.5)	560 (20.1)	1245 (20.7)
	5 (Least deprived)	1720 (19.6)	525 (18.9)	1195 (19.9)
Ethnicity	White	8335 (94.7)	2585 (92.9)	5750 (95.5)
	Asian	35 (0.4)	10 (0.3)	30 (0.5)
	Black	245 (2.8)	100 (3.6)	145 (2.4)
	Mixed	50 (0.6)	35 (1.3)	15 (0.2)
	Other	70 (0.8)	25 (0.9)	45 (0.8)
	Unknown	60 (0.7)	30 (1)	35 (0.6)
Severe liver disease	No	5270 (59.9)	1960 (70.4)	3310 (55)
	Yes	3535 (40.1)	825 (29.6)	2710 (45)
Smoking status	Never	2825 (32.1)	940 (33.8)	1885 (31.3)
	Former	4690 (53.3)	1380 (49.6)	3310 (55)
	Current	1275 (14.5)	460 (16.5)	820 (13.6)
	Unknown[%]	10 (0.1)	redacted (redacted)	redacted (redacted)
Body mass index[$]	Underweight	225 (2.5)	105 (3.7)	120 (2)
	Healthy range	2620 (29.8)	840 (30.1)	1785 (29.6)
	Overweight	2805 (31.8)	815 (29.4)	1985 (33)
	Obese	2285 (26)	740 (26.6)	1545 (25.6)
	Severe obesity	370 (4.2)	120 (4.3)	250 (4.2)
	Unknown	495 (5.6)	160 (5.8)	335 (5.5)
COVID high risk condition	No	7635 (86.8)	2410 (86.7)	5225 (86.8)
	Yes	1165 (13.2)	370 (13.3)	795 (13.2)
UDCA status switched at any point during follow-up		2130 (24.2)	575 (20.7)	1555 (25.8)

Supplementary Table 2: Characteristics of those with PSC by ursodeoxycholic acid (UDCA) exposure status at index date

Characteristic		Overall N=2505	No UDCA at baseline N=1295	UDCA at baseline N=1210
Age category	18 - 40 years	690 (27.5)	400 (31)	285 (23.8)
	41 - 60 years	660 (26.4)	340 (26.3)	320 (26.4)
	61 - 80 years	950 (37.9)	445 (34.3)	505 (41.7)
	>80 years	205 (8.2)	110 (8.3)	100 (8.1)
Sex	Female	1080 (43.1)	560 (43.1)	520 (43.2)
	Male	1425 (56.9)	740 (56.9)	685 (56.8)
Index of multiple deprivation	1 (Most deprived)	375 (14.9)	215 (16.4)	160 (13.3)
	2	405 (16.3)	225 (17.3)	185 (15.2)
	3	535 (21.3)	285 (22)	250 (20.5)
	4	605 (24.2)	290 (22.5)	315 (26)
	5 (Least deprived)	585 (23.3)	280 (21.8)	300 (25)
Ethnicity	White	2265 (90.5)	1155 (89.3)	1110 (91.9)
	Asian	25 (0.9)	10 (0.8)	15 (1.1)
	Black	135 (5.5)	85 (6.6)	50 (4.3)
	Mixed	45 (1.9)	25 (2)	20 (1.7)
	Other	20 (0.7)	10 (0.8)	10 (0.7)
	Unknown	10 (0.5)	redacted (redacted)	redacted (redacted)
Severe liver disease	No	2020 (80.7)	1110 (85.6)	910 (75.3)
	Yes	485 (19.3)	185 (14.4)	300 (24.7)
Smoking status	Never	1410 (56.3)	725 (56.1)	680 (56.5)
	Former	885 (35.4)	440 (34)	445 (36.9)
	Current	180 (7.3)	115 (8.8)	70 (5.6)
	Unknown[%]	25 (1.1)	15 (1.2)	10 (1)
Body mass index[$]	Underweight	85 (3.3)	50 (3.9)	30 (2.7)
	Healthy range	885 (35.3)	460 (35.6)	425 (35)
	Overweight	785 (31.4)	380 (29.2)	405 (33.6)
	Obese	435 (17.3)	215 (16.7)	215 (17.9)
	Severe obesity	55 (2.2)	35 (2.5)	20 (1.7)
	Unknown	265 (10.5)	155 (12)	110 (9)
COVID high risk condition	No	1980 (79.1)	1050 (81.2)	925 (76.8)
	Yes	525 (20.9)	245 (18.8)	280 (23.2)
UDCA status switched at any point during follow-up		585 (5.17)	585 (45.2)	435 (36.0)

Schoenfeld residual plots

Supplementary Figure 2: Primary outcome: COVID-19 related hospitalisation or death

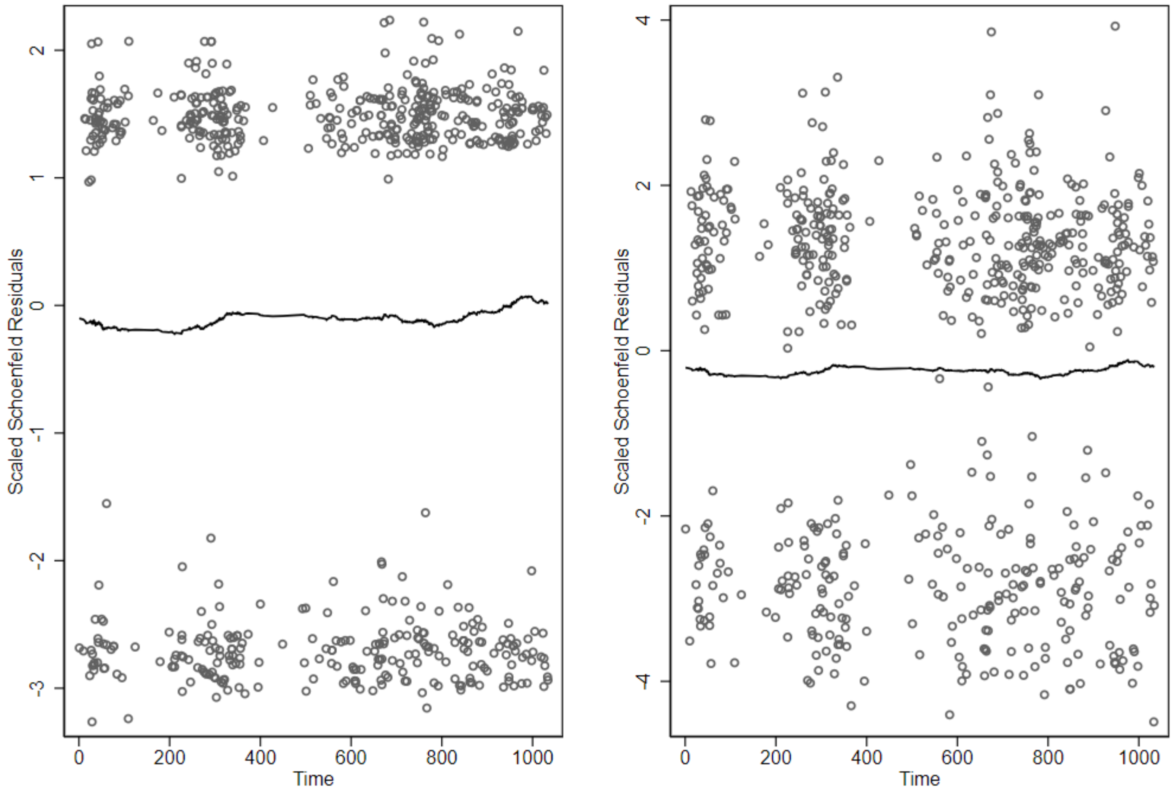

Supplementary Figure 3: Secondary outcome: COVID-19 related hospitalisation

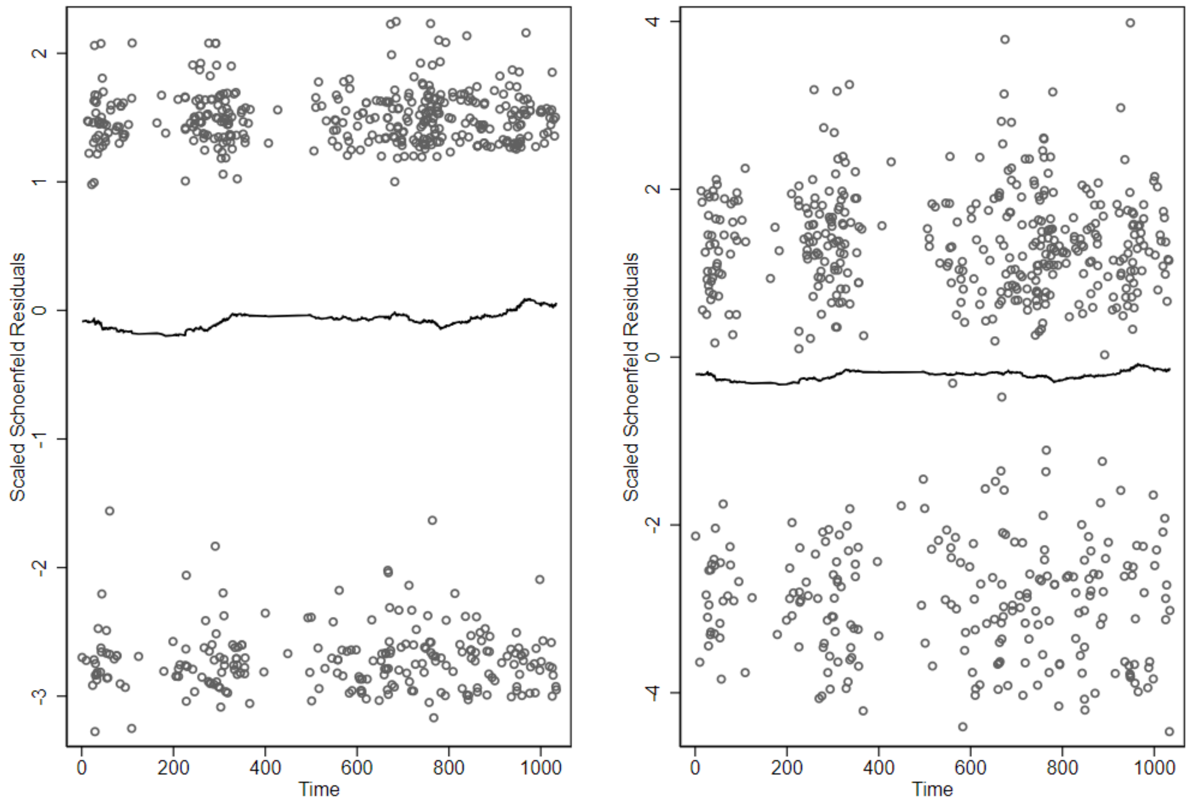

Supplementary Figure 4: Secondary outcome: COVID-19 related death

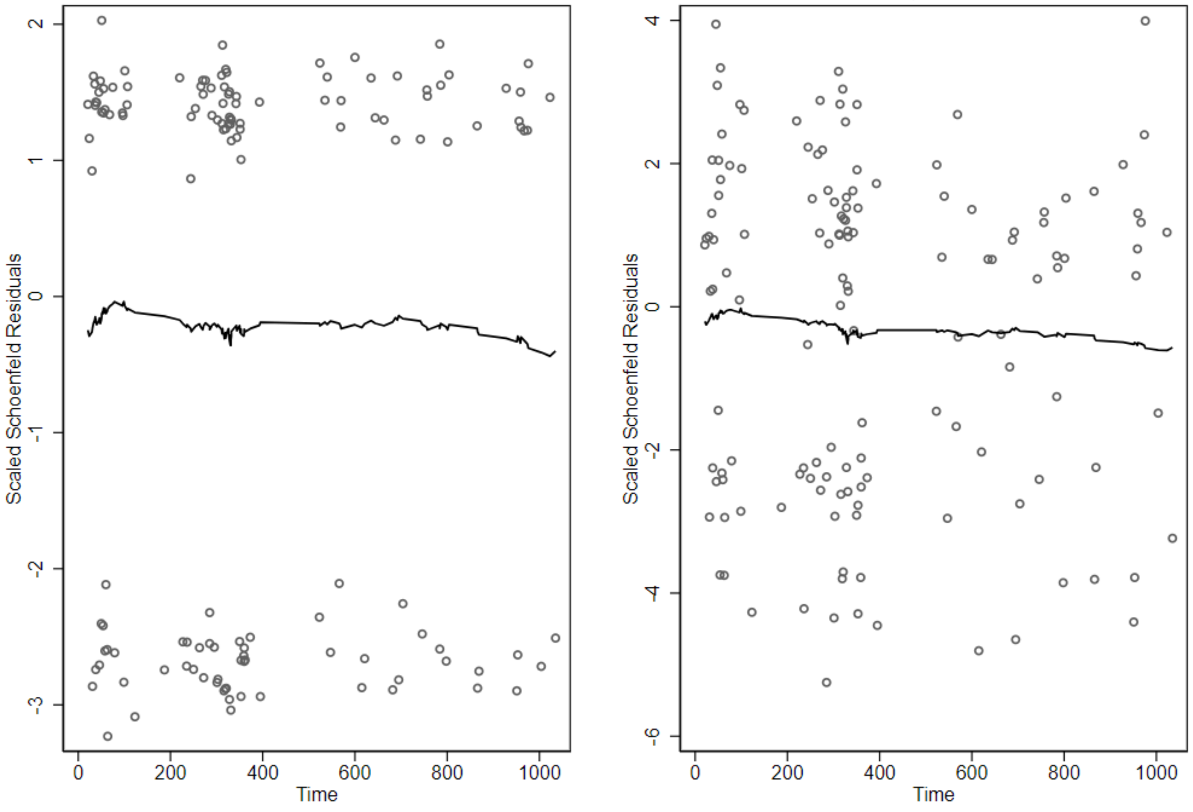

Sensitivity analysis results

Supplementary Table 3: 90 day exposure

Outcome	Exposure status	Events	Rate per 100,000	Unadjusted model Hazard ratio (95% confidence interval)	Age and sex adjusted model Hazard ratio (95% confidence interval)	Adjusted model Hazard ratio (95% confidence interval)
Composite (hospitalisation or death)	No UDCA	250	183.8	Reference	Reference	Reference
	UDCA	390	175.7	0.92 (0.78 - 1.07)	0.88 (0.75 - 1.04)	0.79 (0.68 - 0.93)
COVID-19 related death	No UDCA	60	43.2	Reference	Reference	Reference
	UDCA	80	35.6	0.77 (0.55 - 1.09)	0.81 (0.57 - 1.13)	0.7 (0.49 - 1.00)
COVID-19 related hospitalisation	No UDCA	235	172.8	Reference	Reference	Reference
	UDCA	375	168.9	0.94 (0.80 - 1.10)	0.9 (0.77 - 1.06)	0.81 (0.68 - 0.95)

Supplementary Table 4: 120 day exposure with overlapping days added to next available unexposed period

Outcome	Exposure status	Events	Rate per 100,000	Unadjusted model Hazard ratio (95% confidence interval)	Age and sex adjusted model Hazard ratio (95% confidence interval)	Adjusted model Hazard ratio (95% confidence interval)
Composite (hospitalisation or death)	No UDCA	230	185.6	Reference	Reference	Reference
	UDCA	415	177.9	0.93 (0.80 - 1.10)	0.91 (0.77 - 1.07)	0.81 (0.68 - 0.95)
COVID-19 related death	No UDCA	50	39.6	Reference	Reference	Reference
	UDCA	85	35.8	0.85 (0.60 - 1.19)	0.9 (0.63 - 1.27)	0.79 (0.55 - 1.13)
COVID-19 related hospitalisation	No UDCA	215	173.5	Reference	Reference	Reference
	UDCA	395	168.8	0.96 (0.82 - 1.14)	0.93 (0.79 - 1.10)	0.83 (0.70 - 0.98)

Supplementary Table 5: PBC population only

Outcome	Exposure status	Events	Rate per 100,000	Unadjusted model Hazard ratio (95% confidence interval)	Age and sex adjusted model Hazard ratio (95% confidence interval)	Adjusted model Hazard ratio (95% confidence interval)
Composite (hospitalisation or death)	No UDCA	170	130.3	Reference	Reference	Reference
	UDCA	325	142.8	0.84 (0.70 - 1.01)	0.87 (0.72 - 1.05)	0.79 (0.65 - 0.95)
COVID-19 related death	No UDCA	45	33.8	Reference	Reference	Reference
	UDCA	70	30.4	0.67 (0.46 - 0.98)	0.77 (0.52 - 1.13)	0.68 (0.46 - 1.02)
COVID-19 related hospitalisation	No UDCA	160	122.7	Reference	Reference	Reference
	UDCA	310	136.2	0.88 (0.72 - 1.06)	0.9 (0.74 - 1.09)	0.82 (0.67 - 0.99)

Supplementary Table 6: PSC population only

Outcome	Exposure status	Events	Rate per 100,000	Unadjusted model Hazard ratio (95% confidence interval)	Age and sex adjusted model Hazard ratio (95% confidence interval)	Adjusted model Hazard ratio (95% confidence interval)
Composite (hospitalisation or death)	No UDCA	70	160.7	Reference	Reference	Reference
	UDCA	75	209.4	1.21 (0.87 - 1.68)	1.13 (0.82 - 1.57)	1.02 (0.73 - 1.41)
COVID-19 related death	No UDCA	Redacted				
	UDCA	Redacted				
COVID-19 related hospitalisation	No UDCA	70	160.7	Reference	Reference	Reference
	UDCA	70	195.5	1.19 (0.85 - 1.65)	1.12 (0.80 - 1.56)	0.99 (0.71 - 1.38)

Supplementary Table 7: Exclusion of people prescribed obeticholic acid at baseline

Outcome	Exposure status	Events	Rate per 100,000	Unadjusted model Hazard ratio (95% confidence interval)	Age and sex adjusted model Hazard ratio (95% confidence interval)	Adjusted model Hazard ratio (95% confidence interval)
Composite (hospitalisation or death)	No UDCA	240	184.7	Reference	Reference	Reference
	UDCA	400	178.2	0.92 (0.79 - 1.08)	0.89 (0.76 - 1.05)	0.8 (0.68 - 0.94)
COVID-19 related death	No UDCA	55	41.5	Reference	Reference	Reference
	UDCA	85	37.4	0.82 (0.59 - 1.16)	0.86 (0.61 - 1.22)	0.76 (0.53 - 1.09)
COVID-19 related hospitalisation	No UDCA	225	173.1	Reference	Reference	Reference
	UDCA	380	169.3	0.95 (0.80 - 1.12)	0.91 (0.77 - 1.08)	0.82 (0.69 - 0.97)

Supplementary Table 8: Exclusion of people with missing information for smoking status, BMI and ethnicity.

Outcome	Exposure status	Events	Rate per 100,000	Unadjusted model Hazard ratio (95% confidence interval)	Age and sex adjusted model Hazard ratio (95% confidence interval)	Adjusted model Hazard ratio (95% confidence interval)
Composite (hospitalisation or death)	No UDCA	225	189.7	Reference	Reference	Reference
	UDCA	380	178.2	0.91 (0.77 - 1.07)	0.89 (0.75 - 1.05)	0.79 (0.67 - 0.94)
COVID-19 related death	No UDCA	50	41.3	Reference	Reference	Reference
	UDCA	85	39.4	0.84 (0.59 - 1.19)	0.89 (0.63 - 1.27)	0.78 (0.54 - 1.12)
COVID-19 related hospitalisation	No UDCA	210	177.0	Reference	Reference	Reference
	UDCA	365	171.1	0.93 (0.78 - 1.10)	0.91 (0.76 - 1.07)	0.81 (0.68 - 0.96)

Supplementary Table 9: Secondary analysis: Vaccinated cohort with index date 1st March 2021

Outcome	Exposure status	Events	Rate per 100,000	Unadjusted model Hazard ratio (95% confidence interval)	Age and sex adjusted model Hazard ratio (95% confidence interval)	Adjusted model Hazard ratio (95% confidence interval)
Composite (hospitalisation or death)	No UDCA	170	190.8	Reference	Reference	Reference
	UDCA	250	161.5	0.83 (0.68 - 1.01)	0.79 (0.65 - 0.96)	0.71 (0.58 - 0.87)
COVID-19 related death	No UDCA	20	22.16	Reference	Reference	Reference
	UDCA	25	15.99	0.71 (0.40 - 1.25)	0.7 (0.40 - 1.21)	0.72 (0.40 - 1.32)
COVID-19 related hospitalisation	No UDCA	165	185.14	Reference	Reference	Reference
	UDCA	245	158.29	0.85 (0.69 - 1.03)	0.81 (0.66 - 0.98)	0.73 (0.59 - 0.89)

Supplementary Table 10: OpenSAFELY Collaborative

First name and middle initial	Surname
Alex J	Walker
Brian	MacKenna
Peter	Inglesby
Ben	Goldacre
Helen J	Curtis
Caroline E	Morton
Jessica	Morley
Amir	Mehrkar
Sebastian CJ	Bacon
George	Hickman
Richard	Croker
David	Evans
Tom	Ward
Nicholas J	DeVito
Louis	Fisher
Amelia CA	Green
Jon	Massey
Rebecca M	Smith
William J	Hulme
Simon	Davy
Colm D	Andrews
Lisa EM	Hopcroft
Henry	Drysdale
Iain	Dillingham
Robin Y	Park
Rose	Higgins
Christine	Cunningham
Milan	Wiedemann
Linda	Nab
Steven	Maude
Orla	Macdonald
Ben FC	Butler-Cole
Thomas	O'Dwyer
Catherine L	Stables
Christopher	Wood
Andrew D	Brown
Victoria	Speed
Lucy	Bridges
Andrea L	Schaffer
Caroline E	Walters
Christopher T	Rentsch
Krishnan	Bhaskaran
Anna	Schultze
Elizabeth J	Williamson
Helen I	McDonald
Laurie A	Tomlinson
Rohini	Mathur
Rosalind M	Eggo
Kevin	Wing
Angel YS	Wong
John	Tazare
Richard	Grieve
Daniel J	Grint
Sinead	Langan
Kathryn E	Mansfield
Ian J	Douglas
Stephen JW	Evans
Liam	Smeeth
Jemma L	Walker
Viyaasan	Mahalingasivam
Harriet	Forbes
Thomas E	Cowling
Emily L	Herrett
Ruth E	Costello
Bang	Zheng
Edward P K	Parker
Christopher	Bates
Jonathan	Cockburn
John	Parry
Frank	Hester
Sam	Harper
Shaun	O'Hanlon
Alex	Eavis
Richard	Jarvis
Dima	Avramov
Paul	Griffiths
Aaron	Fowles
Nasreen	Parkes
Brian	Nicholson
Rafael	Perera
David	Harrison
Kamlesh	Khunti
Jonathan AC	Sterne
Jennifer	Quint

Supplementary Table 11: The LH&W NCS Collaborative

First name	Surname
Nishi	Chaturvedi
Chloe	Park
Alisia	Carnemolla
Dylan	Williams
Anika	Knueppel
Andy	Boyd
Emma L	Turner
Katharine M	Evans
Richard	Thomas
Samantha	Berman
Stela	McLachlan
Matthew	Crane
Rebecca	Whitehorn
Jacqui	Oakley
Diane	Foster
Hannah	Woodward
Kirsteen C	Campbell
Nicholas	Timpson
Alex	Kwong
Ana Goncalves	Soares
Gareth	Griffith
Renin	Toms
Louise	Jones
Herbert,	Annie
Ruth	Mitchell
Tom	Palmer
Jonathan	Sterne
Venexia	Walker
Lizzie	Huntley
Laura	Fox
Rachel	Denholm
Rochelle	Knight
Kate	Northstone
Arun	Kanagaratnam
Elsie	Horne
Harriet	forbes
Teri	North
Kurt	Taylor
Marwa AL	Arab
Scott	Walker
Jose IC	Coronado
Arun S	Karthikeyan
George	Ploubidis
Bettina	Moltrecht
Charlotte	Booth
Sam	Parsons
Bozena	Wielgoszewska
Charis	Bridger-Staatz
Claire	Steves
Ellen	Thompson
Paz	Garcia
Nathan	Cheetham
Ruth	Bowyer
Maxim	Freydin
Amy	Roberts
Ben	Goldacre
Alex	Walker
Jess	Morley
William	Hulme
Linda	Nab
Louis	Fisher
Brian	MacKenna
Colm	Andrews
Helen	Curtis
Lisa	Hopcroft
Amelia	Green
Praveetha	Patalay
Jane	Maddock
Kishan	Patel
Jean	Stafford
Wels	Jacques
Kate	Tilling
John	Macleod
Eoin	McElroy
Anoop	Shah
Richard	Silverwood
Spiros	Denaxas
Robin	Flaig
Daniel	McCartney
Archie	Campbell
Laurie	Tomlinson
John	Tazare
Bang	Zheng
Liam	Smeeth
Emily	Herrett
Thomas	Cowling
Kate	Mansfield
Ruth E	Costello
Kevin	Wang
Kathryn	Mansfield
Viyaasan	Mahalingasivam
Ian	Douglas
Sinead	Langan
Sinead	Brophy
Michael	Parker
Jonathan	Kennedy
Rosie	McEachan
John	Wright
Kathryn	Willan
Ellena	Badrick
Gillian	Santorelli
Tiffany	Yang
Bo	Hou
Andrew	Steptoe
Di Gessa,	Giorgio
Jingmin	Zhu
Paola	Zaninotto
Angela	Wood
Genevieve	Cezard
Samantha	Ip
Tom	Bolton
Alexia	Sampri
Elena	Rafeti
Fatima	Almaghrabi
Aziz	Sheikh
Syed A	Shah
Vittal	Katikireddi
Richard	Shaw
Olivia	Hamilton
Michael	Green
Theocharis	Kromydas
Daniel	Kopasker
Felix	Greaves
Robert	Willans
Fiona	Glen
Steve	Sharp
Alun	Hughes
Andrew	Wong
Lee Hamill	Howes
Alicja	Rapala
Lidia	Nigrelli
Fintan	McArdle
Chelsea	Beckford
Betty	Raman
Richard	Dobson
Amos	Folarin
Callum	Stewart
Yatharth	Ranjan
Jd	Carpentieri
Laura	Sheard
Chao	Fang
Sarah	Baz
Andy	Gibson
John	Kellas
Stefan	Neubauer
Stefan	Piechnik
Elena	Lukaschuk
Laura C	Saunders
James M	Wild
Stephen	Smith
Peter	Jezzard
Elizabeth	Tunnicliffe
Zeena-Britt	Sanders
Lucy	Finnigan
Vanessa	Ferreira
Mark	Green
Rebecca	Rhead
Milla	Kibble
Yinghui	Wei
Agnieszka	Lemanska
Francisco	Perez-Reche
Dominik	Piehlmaier
Lucy	Teece
Edward	Parker
