# Supplementary material for: Ursodeoxycholic acid and severe COVID-19 outcomes in a cohort study using the OpenSAFELY platform
Source: Commun Med (Lond). 2024 Nov 19;4:238. doi: 10.1038/s43856-024-00664-y (PMC11576861; doi:10.1038/s43856-024-00664-y)
Supplement: Supplementary file 3 — Reporting summary [file 43856_2024_664_MOESM3_ESM.pdf]

Corresponding author(s): *Double-blind peer review submissions: write DBPR and your manuscript number here instead of author names.*

Last updated by author(s): *YYYY-MM-DD*

Reporting Summary

Nature Portfolio wishes to improve the reproducibility of the work that we publish. This form provides structure for consistency and transparency in reporting. For further information on Nature Portfolio policies, see our Editorial Policies and the Editorial Policy Checklist.

Statistics

For all statistical analyses, confirm that the following items are present in the figure legend, table legend, main text, or Methods section.

n/a	Confirmed	
☐	☒	The exact sample size (*n*) for each experimental group/condition, given as a discrete number and unit of measurement
☐	☒	A statement on whether measurements were taken from distinct samples or whether the same sample was measured repeatedly
☐	☒	The statistical test(s) used AND whether they are one- or two-sided<br>*Only common tests should be described solely by name; describe more complex techniques in the Methods section.*
☐	☒	A description of all covariates tested
☐	☒	A description of any assumptions or corrections, such as tests of normality and adjustment for multiple comparisons
☐	☒	A full description of the statistical parameters including central tendency (e.g. means) or other basic estimates (e.g. regression coefficient) AND variation (e.g. standard deviation) or associated estimates of uncertainty (e.g. confidence intervals)
☐	☒	For null hypothesis testing, the test statistic (e.g. *F*, *t*, *r*) with confidence intervals, effect sizes, degrees of freedom and *P* value noted<br>*Give P values as exact values whenever suitable.*
☒	☐	For Bayesian analysis, information on the choice of priors and Markov chain Monte Carlo settings
☐	☒	For hierarchical and complex designs, identification of the appropriate level for tests and full reporting of outcomes
☐	☒	Estimates of effect sizes (e.g. Cohen's *d*, Pearson's *r*), indicating how they were calculated

Our web collection on statistics for biologists contains articles on many of the points above.

Software and code

Policy information about availability of computer code

Data collection	Data management was performed using Python 3.8, with analysis carried out using Stata 17. Code for data management and analysis as well as codelists available online (https://github.com/opensafely/udca_covid).
Data analysis	Data management was performed using Python 3.8, with analysis carried out using Stata 17. Code for data management and analysis as well as codelists available online (https://github.com/opensafely/udca_covid).

For manuscripts utilizing custom algorithms or software that are central to the research but not yet described in published literature, software must be made available to editors and reviewers. We strongly encourage code deposition in a community repository (e.g. GitHub). See the Nature Portfolio guidelines for submitting code & software for further information.

Data

Policy information about availability of data

All manuscripts must include a data availability statement. This statement should provide the following information, where applicable:

- Accession codes, unique identifiers, or web links for publicly available datasets
- A description of any restrictions on data availability
- For clinical datasets or third party data, please ensure that the statement adheres to our policy

All data were linked, stored and analysed securely within the OpenSAFELY platform https://opensafely.org/. All code is shared openly for review and re-use under MIT open license. Detailed pseudonymised patient data is potentially re-identifiable and therefore not shared.

Human research participants

Policy information about studies involving human research participants and Sex and Gender in Research.

Reporting on sex and gender	We report only sex as this is what is recorded in the electronic health records.
Population characteristics	We describe age, sex, ethnicity, deprivation, sustainability and transformation partnership (STP) region (an NHS administrative geographical area), body mass index, smoking status, and presence of a COVID-19 high-risk diagnosis (i.e., learning difficulties, solid cancer, haematological disease, stem cell transplant, renal disease, immune-mediated inflammatory disorders identified through immunosuppressant drugs and glucocorticoid prescribing, primary immune deficiencies, HIV/AIDS, solid organ transplant, or rare neurological conditions including multiple sclerosis, motor neurone disease, myasthenia gravis or Huntington's disease).
Recruitment	N/A study population identified through electronic health records.
Ethics oversight	This study was approved by the Health Research Authority (REC reference 20/LO/0651) and by the LSHTM Ethics Board (reference 21863).

Note that full information on the approval of the study protocol must also be provided in the manuscript.

Field-specific reporting

Please select the one below that is the best fit for your research. If you are not sure, read the appropriate sections before making your selection.

☒ Life sciences ☐ Behavioural & social sciences ☐ Ecological, evolutionary & environmental sciences

For a reference copy of the document with all sections, see nature.com/documents/nr-reporting-summary-flat.pdf

Life sciences study design

All studies must disclose on these points even when the disclosure is negative.

Sample size	There were 11,305 people with of primary biliary cholangitis or primary sclerosing cholangitis included in the study.
Data exclusions	People were excluded if they had: 1) <18 or >115 years of age, 2) < 6 months of registration in a TPP practice at the index date, which could preclude adequate ascertainment of key covariates, 3) missing information on sex, sustainability and transformation partnership (STP) region (an NHS administrative geographical area) or index of multiple deprivation (IMD), likely indicative of poor data quality or 4) a liver transplant prior to the index date, identified using SNOMED-CT codes in the primary care records or OPCS codes in secondary care records.
Replication	Results were not replicated within this study, however code has been shared so other people could replicate the study with their data.
Randomization	Treatments were not assigned randomly. Instead, treatment groups were defined by prescribing records, confounders were then adjusted for in analysis.
Blinding	Not relevant as this is an observational study using routinely collected data.

Reporting for specific materials, systems and methods

We require information from authors about some types of materials, experimental systems and methods used in many studies. Here, indicate whether each material, system or method listed is relevant to your study. If you are not sure if a list item applies to your research, read the appropriate section before selecting a response.

Materials & experimental systems

n/a	Involved in the study	
☒	☐	Antibodies
☒	☐	Eukaryotic cell lines
☒	☐	Palaeontology and archaeology
☒	☐	Animals and other organisms
☒	☐	Clinical data
☒	☐	Dual use research of concern

Methods

n/a	Involved in the study	
☒	☐	ChIP-seq
☒	☐	Flow cytometry
☒	☐	MRI-based neuroimaging
